# Targeting Fatigue in Stroke Patients

**DOI:** 10.5402/2011/805646

**Published:** 2011-11-30

**Authors:** Andrew W. Barritt, David G. Smithard

**Affiliations:** ^1^Division of Medicine, Royal Sussex County Hospital, Brighton, East Sussex BN2 5BE, UK; ^2^Kent Community Health NHS Trust, Ashford, Kent TN24 5AZ, UK

## Abstract

Symptoms of fatigue are often reported by patients in both the acute and chronic stages of recovery following a stroke. It is commonly associated with low mood and sleep disturbances, but can arise in their absence. However, it has also been associated with poorer long-term outcome and, as such, its aetiology warrants a greater understanding. There is convincing evidence that inflammatory cascades and cytokine signalling precipitated by the infarct promote fatigue, and these pathways may harbour therapeutic targets in its management.

## 1. Introduction

The repercussions of stroke can be devastating, given that it can suddenly and permanently impair some of the most basic functions we all take for granted, including our mobility, speech, and effective swallowing [1]. A national campaign by The Stroke Association [2], termed “Act F.A.S.T.”, has recently promoted the swift presentation to healthcare services for any patient suspected of having acute stroke, with assessment for possible early thrombolysis. Gold standard care in hospital involves admission to a designated stroke unit. Here, a battery of investigations are performed to maximise secondary prevention, and a multidisciplinary team is able to coordinate timely rehabilitation and attention to ongoing medical, nutritional, and psychological needs. Amongst the risks of developing anxiety states and disorders of (usually low) mood, there exists a frequently occurring symptom of poststroke fatigue (PSF) which can blight stroke patients' recovery long after their physical disabilities have resolved. 

Within the nervous system, fatigue can be centrally or peripherally mediated [3]. The latter, for instance, is seen in neuromuscular junction disorders, such as myasthenia gravis, and does have definable parameters as a clinical sign—these being diminished postexertional muscle power on the Medical Research Council (MRC) scale and a 10% decrement of motor end-plate potentials on electromyography recordings. However, central fatigue is not so easily defined and symptomatically lies on a continuous spectrum encompassing a whole host of fluid terms including vitality, motivation, and mood. It has been described in multiple sclerosis patients as “a subjective lack of physical and/or mental energy that is perceived by the individual or caregiver to interfere with usual or desired activities” [4] and is thus, by virtue of its own broad definition, a rather idiosyncratic experience open to personal interpretation. This is reflected by the large number of assessment scales or scoring systems for fatigue to be found throughout the literature. To name a few, there are linear analogue scales (visual analogue scale, VAS [5–8]) and questionnaire-based systems (vitality domain in the Short-Form 36, VIT SF-36 [9, 10]; fatigue assessment scale, FAS [11]; checklist individual strength, CIS [12]; fatigue severity scale, FSS [8, 13–16]; modified fatigue impact scale, MFIS [17]) which do make it impossible to standardise the data on fatigue. Nevertheless, despite PSF research being comparative in its infancy, some important studies have been conducted to help characterise the mechanisms of onset and provide some understanding of methods to diminish or, at least, control the symptoms for patients.

## 2. Poststroke Fatigue and Its Associations

The symptom of fatigue following a stroke is commonly found and has been described as one of the most difficult to cope with [18]. The negative impact of PSF can be felt irrespective of whether the patient is at the younger [19] or older [20] end of the spectrum as it hampers their attempts to return to normality and regain independence. It is tricky to state a precise prevalence given that multiple studies have assessed patients at very wide-ranging times since their stroke onset, and each with different measurements of fatigue. However, at least 30% of patients, if not double that, will experience it sometime in their recovery. It can be present within weeks [15] and persist for many months [14, 21] or even years [6, 10, 13, 22] afterwards. Lerdal and colleagues showed that within two weeks of stroke, 24% of patients had severe fatigue on the FSS, 33% moderate, and 43% mild or none [15]. They reported that greater fatigue was related to a poorer physical function and symptoms of depression, with a similar correlation being reported at one year after stroke supported by Appelros [21]. However, as time progresses, the association with the level of disability becomes disputed [12, 13]. In addition, patients who suffered with premorbid fatigue had an increased chance of PSF [15]; the risk elsewhere was quoted at over 60% [8]. 

The association of PSF to the size of the infarct has not been shown [10], but the site of the lesion may be relevant. It has been suggested that lacunar infarcts located within the basal ganglia, internal capsule [14], and infratentorial areas [12] have been linked to an increased occurrence of PSF, although there is no systematic review available as yet on this. Interestingly, reduced thickness of the posterior parietal cortex in patients with multiple sclerosis has been associated with fatigue scores but, specifically, white matter lesions were not [17]. Mead and coworkers, following people up for 64 months, did not find an association between PSF and stroke location but instead found increased PSF associated with female gender and older age [10]. Harbison et al. were unable to demonstrate any such age or gender association [16], whereas Snaphaan and colleagues found that older age may even be protective against fatigue following stroke [12]. 

There are clearly a lot of conflicting findings in the literature as regards risk factors for PSF. One rather less controversial association with PSF is low mood [10, 12–14, 21–23]. Snaphaan and co-workers used the CIS to show that fatigue at both two months and 18 months after stroke was present in roughly one-third of patients (frequently the same people) and had a statistically significant association with scores on the hospital anxiety depression scale [12]. Interestingly, there was no residual physical impairment on indices of functional status leading the authors to surmise that the PSF may be more psychological than physical. Similarly, Park et al. found that at thirty-two months after stroke there was a positive correlation between the 30% of subjects who were fatigued on the FSS and the 55% with symptoms of depression on a self-report questionnaire, but again there was no convincing relationship to functional impairment [13]. It is recognised that aspects of fatigue and depression can be difficult to tease apart as mood assessment scales often include questions on energy levels and fatigue, and vice versa. It also depends on where the cut-off is taken. Tang et al. accordingly reported increased geriatric depression scale scores in fatigued patients (also assessed by the FSS), despite the fact that patients fulfilling the official DSM-IV diagnosis of depression were excluded from their cohort [14]. Glader et al. showed a strong relationship between constant tiredness and always being depressed [22], and it is difficult to know whether there is some causality. Nevertheless, when patients who “often” and “always” felt depressed were removed from analysis, PSF was still a predictor for worse outcome, including more dependence in activities of daily living and poorer general health [22]. It was also a predictor for death, again independently of depression. With the potential for PSF to have implications for mortality as well as morbidity, it is vital for us to further understand the possible mechanisms of fatigue so that effective management strategies can be devised.

## 3. Mechanisms of Fatigue

Fatigue does not only have a physical basis, but also comprises mental and psychological aspects [18] which can all coexist in the same patient. It may be that merely utilising executive thinking to plan certain activities gives rise to a feeling of fatigue and rapid exhaustion [23]. Although it is clear low mood and, indeed, the degree of functional recovery do not necessarily account for a sizable proportion of PSF, treating depressive symptoms may make a valuable difference to the emotional state and motivation of the patient. In a study where all the patients had PSF on two assessment scales, the selective serotonin reuptake inhibitor antidepressant fluoxetine failed to make any change to the fatigue scores after three months' therapy, yet dutifully improved poststroke depression and emotional disturbance [8]. Therefore, other factors precipitated by stroke must be responsible for PSF. 

Stroke is not alone in being accompanied by fatigue symptoms. Acute or chronic infections, long-term autoimmune conditions such as rheumatoid arthritis or systemic lupus erythematosus, as well as malignant entities, particularly following oncological treatments, are commonly associated with fatigue. Even multiple sclerosis, which selectively affects the central nervous system (CNS), is notorious for its association with fatigue. The common denominator is activation of an inflammatory response. Inflammation precipitates secretion of various cytokines necessary for immune signalling including interleukin-6 (IL-6), interleukin-1 beta (IL-1*β*), and tumour necrosis factor alpha (TNF*α*) all of which have sites of action within the central nervous system [24]. They are thought to induce the so-called “sickness behaviour” of decreased mood, lowered libido, poor appetite, sleep disturbances, psychomotor slowing, and, importantly, fatigue [25, 26]. These attributes are considered evolutionarily advantageous for overcoming injury and illness and, ordinarily, would resolve once the insult has passed. However, in situations where there is ongoing activation the effects are accordingly continued. Studies on cancer patients who have chronic exposure to TNF*α* over time show that two syndromes of sickness behaviour emerge: an early onset but persistent “neurovegetative” state encompassing fatigue, sleep problems, and psychomotor slowing followed later by an additional “mood and cognitive” state in which there are depression, anxiety, and impairment of memory and attention [25, 27, 28]. The latter syndrome tends to be the one which responds to antidepressants whereas the former does not [25] and might explain why fluoxetine did not affect chronic PSF but did improve mood [8]. 

Acute stroke similarly causes secretion of cytokines, chemokines, and proteases from activated microglia at the infarct epicentre accompanied by release of cytotoxic-free radicals [29]. There is also early upregulation of Toll-like receptors (TLRs) in neurons, which may be involved in proapoptotic pathways, as well as in neighbouring microglia and astrocytes [30]. Cascades downstream of these receptors enable the propagation of inflammation with production of further cytokines [31], and it may be that a greater response is mounted in older patients [25] for whom the majority of strokes will befall. The components of sickness behaviour, including fatigue, are then believed to be mediated through neural, immune, and endocrine mechanisms. Dopaminergic and serotonergic neurotransmitter systems are thought to be affected, with increased expression of their uptake transporters, interference with their synthesis, and possibly altered numbers of receptors [25]. Based on data collected in studies of exercise-induced fatigue, it has been proposed that it is the relative predominance of serotonin compared to dopamine which precipitates fatigue [32, 33], and that exercise training increases plasticity of dopaminergic circuitry leading to a more delayed onset over time. Cytokines may also promote excitotoxic processes involving glutamatergic neurotransmission causing cell death, blood brain barrier breakdown, and impaired homeostasis of astrocyte and neuron metabolism [25, 34]. Production of neurotrophins, such as brain-derived neurotrophic factor (BDNF) which is important for synaptic plasticity and neuron survival both in times of health and following nervous system injury, may be reduced [25]. A lack of BDNF may underlie the disorders of psychomotor slowing and memory disturbance particularly given that hippocampal functions rely heavily on its availability [35]. Moreover, dopamine-secreting neurons involved in movement generation also widely express BDNF and its receptor, TrkB. Both of these are upregulated within the CNS by voluntary exercise and may underlie the postponement of fatigue onset as mentioned above [32]. 

The hypothalamic-pituitary-adrenal (HPA) axis and the autonomic nervous system, which enable communication between the CNS and endocrine systems, oversee glucocorticoid and catecholamine production, respectively, in times of stress, and their activity can be modulated by cytokines as interleukin receptors can be found throughout the HPA axis and afferent vagal ganglia [25, 26]. Short-term hyperactivity of the HPA may be achieved in the acute phase, but after prolonged stimulation, there is a blunting of the normal diurnal cortisol secretion curve with reduced glucocorticoid production and onset of fatigue and depressive symptoms [25, 26]. Hypoactivity of the HPA axis owing to decreased corticotrophin releasing hormone has been accordingly found in patients with the chronic fatigue syndrome (CFS) as well as in chronic autoimmune conditions [36]. Cortisol normally has a negative feedback role on the sympathetic nervous system and on inflammation, promoting humeral immune responses in preference to cytokine production [25, 26]. Disordered sympathetic control, therefore, results and may underpin the observations by Harbison and colleagues that systemic hypertension above 145/90 mmHg and diastolic dips below 50 mmHg on ambulatory blood pressure monitoring in chronic stroke patients is associated with PSF [16]. Again this has been seen also in patients with CFS [37]. 

IL-6 is associated with higher scores of fatigue [38] and, when measured peripherally during the first week after stroke, its peak level correlates positively with infarct volume, white blood cell count, and the acute phase response C-reactive protein (CRP) and negatively with functional outcome and mortality at 1 year [39]. Elevated CRP has itself been associated with higher levels of PSF, particularly when patients with depression were removed from the analysis, although statistical significance was not achieved [11]. It is additionally raised in patients suffering with evident and subclinical CFS, although it is acknowledged that CRP may not be the best surrogate biomarker of CNS inflammation as it is not specific to the condition [40]. Concurrent infections are common in stroke victims and will affect the blood CRP level. Nevertheless, inflammatory signalling is highly likely to play a central part in generating PSF along with other forms of fatigue, given that IL-6 is raised in cancer [38] and CFS [41] patients too. 

Disorders of sleep have been mentioned as part of sickness behaviour [25, 26] and are commonly seen after stroke. They have also been linked to PSF [9, 13, 21] which may be improved when sleep issues are corrected [42]. Park and co-workers found that patients' FSS score correlated significantly with subjective sleeping problems like insomnia or frequent waking [13]. A year or more after suffering a stroke causing only upper limb deficits, half of patients said they were more sleepy than normal during daylight hours and slept for longer periods at night, although the latter tended to be less of an issue as time from stroke increased [9]. In addition, their sleepiness was unrelated to depression or external stresses. It would be important to rule out an obstructive sleep apnoea, the aetiology of which may be entirely unrelated to stroke, but universal use of continuous airway pressure certainly does not resolve PSF when it is absent [43]. In fact poststroke sleep disorders and PSF may have their origins not only in cytokine signalling but perhaps also in the disruption of certain neural networks as a direct consequence of the infarct. Staub and Bogousslavsky have proposed that the ascending reticular activating system concerned with maintaining tonic attention may become damaged by brain stem and subcortical lesions leading to PSF [18] which was inferred in the study by Tang and co-workers mentioned earlier [14]. Nevertheless, some of the putative mechanisms of PSF discussed provide a basis for the investigation of effective interventions in stroke patients.

## 4. Interventions for Poststroke Fatigue

A systematic review in 2008 concluded that insufficient evidence existed to recommend any single treatment for PSF [44] and, to date, there continue to be no published guidelines. However, management guidelines for CFS have been available from the National Institute for Health and Clinical Excellence for several years [45] and may well be applicable to PSF sufferers. Whereas the diagnosis for CFS relies on there being new onset fatigue for at least four months with no identifiable cause, we have discussed the overlap of symptoms between the two conditions, as well as the possible mutual roles of cytokine and inflammatory signalling in their generation. 

The CFS guideline emphasises the need for patients to recognise their limitations and set realistic goals for improvement, with assistance from graded exercise for aerobic training and cognitive behavioural therapy (CBT) [45]. Indeed, several studies have shown a relationship between poor physical stamina and PSF [6, 7, 46], and aerobic fitness, as measured by peak oxygen consumption (VO_2_), is often reduced in stroke survivors [47]. Lewis and colleagues reported that extensor power of the unaffected lower limb in fully ambulatory chronic stroke patients was inversely proportional to their level of PSF although, in this instance, VO_2_ was not found to correlate with PSF [46]. Tseng and Kluding, conversely, did find a negative correlation between PSF and VO_2_ and that motor control particularly was an independent predictor of PSF [6]. A preliminary trial of a 12-week treatment termed “COGRAT” (Cognitive and Graded Activity Training), which combines education, CBT, and increasing exercise programmes, has demonstrated a significant reduction of fatigue scores for patients with severe PSF [7]. The authors recognise that a certain level of cognitive capability and physical aptitude is necessary to participate in COGRAT but that it fosters a more effective coping strategy with which patients can manage their fatigue [7]. A full clinical trial is currently underway. Nevertheless, it is clear that maximising cardiovascular fitness appropriately is to be encouraged, in addition to identifying other modifiable factors such as depression, sleep apnoea, concurrent infective illness, or biochemical abnormality which could be exacerbating PSF. 

The contribution of pharmacotherapy for PSF is largely unexplored and, as such, none are licensed for use in these circumstances. One of the few drugs to be included in a randomised controlled trial for PSF [44] has been the selective serotonin reuptake inhibitor fluoxetine but, as outlined above, it improved depressive symptoms but not fatigue scores [8]. Indeed, with a high serotonin to dopamine ratio being implicated in central fatigue [32], enhancement of dopaminergic neurotransmission and BDNF availability might be more appropriate targets. The free concentration of the amino acid precursor to serotonin, tryptophan, is believed to be increased in the blood during exercise and may contribute to central serotonin predominance and development of fatigue [48]. Data in healthy subjects suggests that administering more branched-chain amino acids during exercise, and thus more competition for tryptophan uptake, can prolong fatigue onset [48]. Interference with other neurotransmitters, such as GABA with use of the GABA_B_ receptor agonist baclofen, seems to have a similar effect on fatigue onset [32] and may thus help symptoms of PSF. The CNS stimulant modafinil, which is currently approved in narcolepsy and sleep apnoea, has also been piloted in a small number of PSF and fatigued multiple sclerosis patients but only seemed to benefit the latter and those with lacunar infarcts of the brainstem or thalamus [42]. Moreover, a quarter of the participants dropped out due to side-effect intolerance. Studies using melatonin are encouraging but inconclusive [49]. A broad scope for further work trialling these and other drugs specifically for PSF is, therefore, promising. 

Perhaps the most intriguing possibility for treating PSF is agents which can attenuate ischaemic neuro-inflammation and the development of the sickness behaviour syndromes. IL-1ra is a competitive antagonist of the IL-1 receptor which is synthesised endogenously and blocks IL-1 signalling [50]. Administered peripherally in rodent models of middle cerebral artery ischaemia, IL-1ra traverses the blood-brain barrier to protect neurons from excitotoxic cell death and reduced inflammatory damage from microglial activation [51]. Importantly, it also improves functional recovery. Emsley and co-workers have shown that a 72-hour infusion of recombinant human (rh)IL-1ra is safe for use in patients and, when commenced within six hours of acute stroke, leads to reduced levels of IL-6, CRP, and white blood cell count in the days afterwards along with lower disability scores compared to placebo at three months [50]. Although rh1L-1ra was associated with a slight increase in bacterial infections, none of these were serious. No mention was made of fatigue levels in these patients but the study was powered really only for profiling safety. A suitably powered phase 3 study may be able to provide supplementary data on PSF. 

Activated protein C (APC) is another potentially emerging treatment for victims of stroke by virtue of its neuroprotective, anticoagulant, and anti-inflammatory properties and has the inherent ability to cross the BBB [52]. Its effects of particular significance are that it reduces microglial activation and suppresses the production of nuclear factor kappa B (NF-*κ*B) on which the subsequent expression of 1L-1*β*, 1L-6, and TNF*α* relies [53]. If PSF is indeed mediated by inflammatory signalling, APC would hold great potential for fatigue prevention as well as minimising functional deficits after stroke. Versions of recombinant APC can be synthesised which possess diminished anticoagulant properties whilst retaining their neuroprotective and anti-inflammatory ones [52].

## 5. Conclusion

Fatigue after stroke is a common phenomenon which may have a pathological basis resulting from the infarct, but not dependent on its size or degree of functional loss. It is associated the least controversially with low mood and sleep disturbances, but can arise in their absence. It is also associated with poorer long-term outcome and it is, therefore, essential for physicians in both primary and secondary care to be able to recognise PSF. There is convincing evidence that inflammatory cascades and cytokine signalling affect the neural, immune, and endocrine systems thereby precipitating fatigue symptoms, and these pathways may, therefore, provide multiple targets for interventions aimed at reducing PSF.
